# Validity, reliability and feasibility of a new observation rating tool and a post encounter rating tool for the assessment of clinical reasoning skills of medical students during their internal medicine clerkship: a pilot study

**DOI:** 10.1186/s12909-020-02110-8

**Published:** 2020-06-19

**Authors:** Catharina M. Haring, Claudia C. R. Klaarwater, Geert A. Bouwmans, Bernadette M. Cools, Petra J. M. van Gurp, Jos W. M. van der Meer, Cornelis T. Postma

**Affiliations:** Radboud university medical center, Nijmegen, The Netherlands

**Keywords:** History taking, Clinical reasoning

## Abstract

**Background:**

Systematic assessment of clinical reasoning skills of medical students in clinical practice is very difficult. This is partly caused by the lack of understanding of the fundamental mechanisms underlying the process of clinical reasoning.

**Methods:**

We previously developed an observation tool to assess the clinical reasoning skills of medical students during clinical practice. This observation tool consists of an 11-item observation rating form (ORT). In the present study we verified the validity, reliability and feasibility of this tool and of an already existing post-encounter rating tool (PERT) in clinical practice among medical students during the internal medicine clerkship.

**Results:**

Six raters each assessed the same 15 student-patient encounters. The internal consistency (Cronbach’s alfa) for the (ORT) was 0.87 (0.71–0.84) and the 5-item (PERT) was 0.81 (0.71–0.87). The intraclass-correlation coefficient for single measurements was poor for both the ORT; 0.32 (*p* < 0.001) as well as the PERT; 0.36 (*p* < 0.001). The Generalizability study (G-study) and decision study (D-study) showed that 6 raters are required to achieve a G-coefficient of > 0.7 for the ORT and 7 raters for the PERT. The largest sources of variance are the interaction between raters and students. There was a consistent correlation between the ORT and PERT of 0.53 (*p* = 0.04).

**Conclusions:**

The ORT and PERT are both feasible, valid and reliable instruments to assess students’ clinical reasoning skills in clinical practice.

## Background

Assessment of clinical reasoning of medical students in clinical practice, is a complicated and tricky process. There is no consensus on what clinical reasoning exactly comprises and what the driving forces are that determine the process [1].

In clinical practice the performance of students is profoundly influenced by context and content specificity of the clinical problems involved. Large inter-rater differences are known to exist, which are mostly due to different frames of reference of the clinical assessors [2]. Therefore it is generally accepted, that in workplace-based assessments, one should not rely on a single measurement to come to a robust conclusion. Only repeated assessments can yield reliable outcomes [3, 4]. If repeated assessment of clinical reasoning could be done within a framework of pre-specified carefully defined objective criteria, the subjectivity of the assessors might be corrected enough to make the assessment more reproducible and reliable.

In clinical practice, assessment of clinical reasoning involves either direct observation of a clinical encounter between a student and a patient (either live or video recorded) or an assessment of an oral or written report after completion of such an encounter. Both methods have their advantages and disadvantages. Observation takes time, which has an inhibitory effect in the clinical setting. On the other hand it is a very powerful method for targeted feedback [5]. Assessment of an oral or written report can be less time consuming. Students can explain their analysis and interpretation, but essential information about the data-gathering ability or the diagnostic reasoning during the encounter can easily be missed.

For assessment of a student’s clinical reasoning after an encounter, tools already exist. An example is the post-encounter form for clinical reasoning by Durning et al. [6] This form is used to assess a predefined free text post-encounter form that is used by students. Validity, reliability and feasibility is tested in an objective structured clinical examination (OSCE) setting but had not been tested in a setting with real patient encounters.

For assessment of clinical reasoning during observation of an encounter in clinical practice, we found no formats that were analyzed for validity and reliability. Of course, there is experience with residency training, during which assessment of clinical reasoning in clinical practice is often incorporated in mini clinical evaluations or a related single work-based encounter assessment instrument. The validity and reliability of many of these instruments in basic medical education is often not properly established and clinical reasoning is mostly only superficially itemized in these instruments [7]. This makes them less suitable for more in-depth exploration of clinical-reasoning abilities of students and trainees in the clinical phase of their training.

The lack of valid and reliable tools on this subject led us to develop a new 11 item observation rating tool (ORT) for assessment of clinical reasoning by medical students in clinical practice. Our first step in the creation of such a tool was the definition of the phenomena that reveal the clinical-reasoning process of the student [8]. The main indicators of clinical reasoning ability abstracted from students’ behavior that we identified were: taking control, recognizing and responding to relevant information, specifying symptoms, asking specific questions pointing to pathophysiological thinking, placing questions in a logical order, checking agreement with patients, summarizing and body language.

Unanswered is the question how reliable, valid and feasible these criteria are if used in an observation tool in clinical practice. This question remains also unclear for the post-encounter assessment tools in clinical practice.

Hence our research question was: What is the validity and reliability and feasibility of both a new observation rating tool and an existing post-encounter rating tool for assessment of clinical reasoning in the practice setting for medical students during their internal medicine clerkship?

## Methods

### Setting and participants

The study was done in a clinical setting, at the Department of Internal Medicine in our University Medical Centre. The outpatient clinic of Internal Medicine has two consulting rooms equipped with a fixed two camera set-up to be used for educational purposes which were used in this study. Video-observation and feedback are a standard part of medical training during the clerkship. The clerkship of internal medicine is the first clinical clerkship in the first master year (M1).

Participating students were asked to record the history taking of their meetings with real patients. They were asked to start doing this from the third patient they would see. The first meetings could be used to get accustomed to the process of the clinical practical setting and the contacts with real patients. As common practice, students were not accompanied by their supervisor during the encounter with the patient but received feedback from the supervising physician directly after the consultation during the presentation of the patient. Thereafter, the student and supervisor would see the patient together. After the encounter, the supervisor registered among other clinical criteria, a global rating for the clinical reasoning in this case, as a grade from 1 to 10 in accordance to standard practice.

For this study, the students would complete an extra activity; the completion of a post-encounter form (PEF). This was done after history taking and physical examination, before receiving feedback of their supervising physician. Students would send their completed PEF digitally to the researchers. The assessors would afterwards observe and rate the history taking and rate the PEF based on their observation of the video registration and would then complete the ORT and the PERT of this patient-student interaction.

Participating assessors involved in assessment of the students by means of the ORT and PERT, were six principal lecturers, i.e., clinicians with degrees and assignments in medical education. They were not involved as the supervising physicians of the students at the time the recordings were made. They assessed the recordings of the students at the time of their choosing. These assessors received a short instruction about the two rating tools. They were asked to observe the recordings as long as they deemed needed to complete the observation rating tool (ORT). After completing this form, they also completed the post-encounter rating tool (PERT). The time needed to fulfil the assessment was registered by the participants.

### Materials

The observation rating tool (ORT) was developed in a previous qualitative research project [8]. It consisted of 11 pairs of opposite statements about student behaviour related to clinical reasoning. On 5-point scale participants could rate which of the opposite statements was most applicable. (additional file 1).

The student post- encounter form (PEF) and the PERT were modified after Durning et al. [6]. (additional file 2 & 3) This PEF the students had to complete was made up of items capturing the essential parts of clinical reasoning; summary statement, problem list, differential diagnosis, most likely diagnosis with foundation for the most likely diagnosis. A physical examination plan was added. Assessment of these 5 items by the assessors was done by means of the PERT that includes a 5-point Likert-scale for the mentioned elements.

### Case selection

Twenty students were invited to participate in the study. One case per student was used. The first case that did not meet the exclusion criteria was selected. Cases were excluded when a student was not well visible or when audio quality was low, or the patient was not able to communicate easily, e.g., because of language barrier. Cases that would not induce clinical reasoning, for example when a patient presented with an established diagnosis, were also excluded. After inclusion of 15 cases, selection was stopped. We calculated that 15 cases and 6 observers were needed to reach a power of > 80% to detect an intra-class correlation of 0.30 for the new observation form [9].

### Measurements

Feasibility was measured as student completion rate and the time the assessors needed to complete both the rating instruments. Assessors were asked about their approval and satisfaction with the instruments. We included the time for completion, because it is a limiting factor in clinical practice.

Validity for both instruments was measured as follows: [10] Co*ntent validity*; For both instruments content validity was measured in two previous studies [6, 8]. *Internal structure*; analysis of internal consistency and Generalizability study to explore factors of variance. *Response process:* This was measured by rater evaluation of both instruments. *Relation with other variables:* This was done by analysing the association between both instruments.

Reliability of the use of the PERT was already analysed in an OSCE [6], but not for assessment in clinical practice. For both rating tools (PERT and ORT) inter-rater reliability was tested and a Generalizability study (G-study) was performed. A G-study is a means of identifying systematic and unsystematic error variation and estimating the contribution of each one.

Since it is known for workplace-based assessment tools that reliability, when using one rater, is often low, a decision study (D-study) to assess how many raters would be needed for each instrument to reach an acceptable reliability was done. To that purpose the information from the G-study is used in the D-study that pinpoints to one particular element. In this way a more precise estimate can be made.

### Statistics

Cronbach’s alpha was used to calculate internal consistency of scales. Inter-rater agreement was computed using the Intra Class Correlation. We used the ‘two-way mixed model’, because assessments will be done by a selected group of assessors, with measures of ‘consistency’, because the assessment will not be used as a pass or fail test. We used ‘single measures’, since one assessor on the work floor usually performs the assessment during the clerkships.

A G-study was conducted to identify various sources of variance. For evaluation of the observation rating tool a two-facet crossed design with six assessors and 11 items was used. For evaluation of the post-encounter rating tool a two-facet crossed design with six assessors and 5 items was used. A relative G coefficient was computed since we were mostly interested in the rank order of the measurement objects rather than consistency in raw scores.

A D-study was performed to forecast changes in G coefficients with alternate levels of facets (assessors and checklist items).

The association between the two rating tools was calculated using Pearson’s correlation coefficient.

We used SPSS version 20 for intra-class correlation and Pearson’s correlation coefficient. Generalizability study was done using the G1 SPSS program [11].

### Ethics

Participation was voluntary for all students. All patients were informed about the study, agreed and signed the informed consent form for recording of history taking for educational purposes. Approval of the institutional ethical review board was not required according to the guidelines of the institutional research board based on the Dutch law (WMO).

## Results

Eighteen out of 20 students agreed to participate in the study. In the study period, 81 new encounters were planned. Thirty recordings were completed including post-encounter form in combination with a qualitative suitable recording of which 15 were selected for the study.

Observation of the student and completing the ORT took on average 32 min. Completion of the PERT took on average 4 extra minutes. Students needed on average 6.5 min to complete the post-encounter form.

The average total score for the ORT was 32.2 (range 25.5–42.8) and for the PERT was 12.8 (8,00–16.6) and the rating of the supervisor 7.38 (range 6.00–8.00). There was a significant correlation between the ORT and PERT of 0.53 (*p* = 0.04), but no significant correlation between the PERT and the supervisor rating or the ORT and the supervisor rating.

### Internal structure

The internal consistency (Cronbach’s alfa) for the 11- item ORT was 0.87 (0.71–0.84) and the 5-item PERT was 0.81 (0.71–0.87).

### Inter-rater reliability

The intra-class-correlation coefficient was poor for both the ORT; 0.32 (*p* < 0.001) as well as the PERT; 0.36 (*p* < 0.001) for a single measurement.

#### G- and D- study

**Fig. 1:**
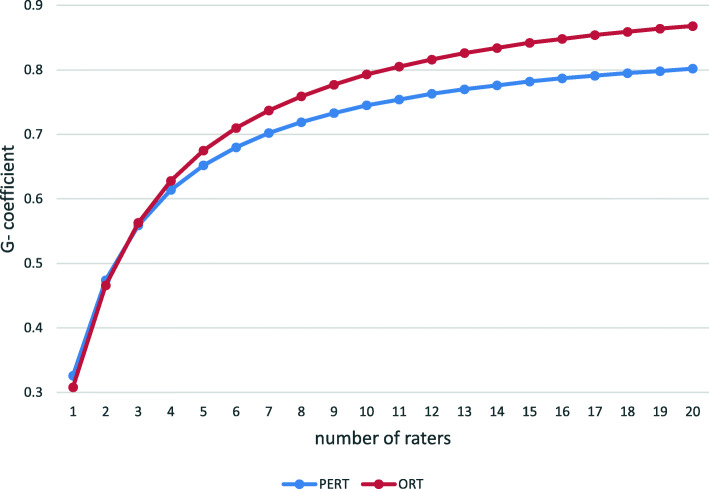
Estimated G-coefficients from the D-study as a function of number of raters per assessment format

The G and D-study (Fig. 1) show that six raters were required to achieve a G-coefficient of > 0.7 for the ORT and seven raters for the PERT. The largest sources of variance (Table 1) were caused by the interaction between raters and students and general sources of error (persons x item x rater) that cannot be further unravelled.

**Table 1:** Variance components from the Generalizability study for the PERT and ORT

	PERT % variance	ORT % variance
Participant	16	12
Item	1.5	5.0
Rater	7.8	4.1
participant x item	12	5.5
participant x rater	24	23
Item x rater	6.5	10
participant x item x rater	33	40

### Response process

Medical students reported that completing the post-encounter form was mostly done after the physical examination for practical reasons. Assessors reported that the assessment procedure was time consuming. The item ‘body language’ in the rating tool was regarded difficult to assess. The assessors all regarded the content of both rating tools comprehensible and adequate.

## Discussion

In this study we developed and evaluated a new instrument, the ORT to assess the clinical-reasoning skills of medical students in the clinical-practice setting. We could demonstrate that the content and construct validity of this instrument were high. We developed the ORT based on the results of a study with experienced clinical teachers [8]. When we compared our instrument with a modified PERT that was developed and proved reliable before [6], we found a significant correlation between the two. However, we found no significant correlation of the global supervisor rating with either the ORT nor the PERT. We interpret this discrepancy as a sign of the poor standardisation and the subjectivity of the traditional global rating of a teacher after a patient encounter.

Also, the inter-rater reliability of both instruments appeared to be poor for a single measurement. These findings are in line with previous studies that investigated the characteristics of work-place based assessment methods [7].

To reach a G-coefficient of > 0.7 with the ORT, 6 raters are required. This is not an uncommon requirement. For example for a tool like the MINI-cex 5 to as many as 60 raters were needed to reach a G-coefficient of 0.7 in different studies [12]. So as already alluded to, one should realize that the assessment of a complicated task such as clinical reasoning cannot not be captured in one observation. Almost all methods to assess proficiency in clinical skills encounter these kinds of problems. As said, a major reason is personal bias of clinical teachers. Even teachers within the same discipline (e.g., internal medicine) and affiliated with the same institution, will vary regarding their opinions on clinical skills. This is because raters make and justify judgments based on personal theories and performance constructs [2].

To attain a fair assessment of medical students, it is worthwhile to discuss these diverse ratings in teach-the-teacher sessions. As this study shows, the low inter-rater reliability of these instruments for a single encounter can be tackled by using more raters assessing the same encounter. In practical terms it is preferable to enhance reliability by having different raters assessing different patient encounters of the same student. Indeed, the latter approach has been found more effective to improve reliability than using one rater to rate different encounters [13].. Several investigators who have evaluated the reliability of clinical assessment methods arrive at this recommendation [14, 15]. An additional advantage is that students will receive feedback on various clinical encounters and problems from different teachers. Ideally, these encounters should deal with a variety of clinical problems to overcome the problem content specificity. In conclusion the ORT and PERT can be used as formative tests for high stakes exams needing 6 to 7 assessors. However, it should preferentially be considered to be used as summative tests by using different clinical encounters and different raters as part of a longitudinal assessment program on clinical reasoning.

The largest source of variance in our study appeared to be the interaction between *person x rater* for both instruments (Table 1). This implies that the ranking order of the students varied greatly between the raters. Unfortunately it is known that rater training has little effect on improvement of outcome of workplace based assessment [16].

It should be noted that the present study used video recordings of clinical encounters and not direct, real-time observation. The obvious reasons for that were of practical nature: in this way the assessors could make the observations at a time and a place that suited them most and the assessor could also be another clinical teacher and not necessarily the supervisor in the clinic.

Next to that real-time observation has the disadvantage that the presence of an observer during the encounter of the student with the patient influences the performance.

Although we have not tested it in this study, it is likely that the ORT instrument can also be used during real-time observations. In further research it should be tested how many cases are needed in that situation to get a reliable assessment of clinical reasoning.

In clinical medicine, most clinicians have a tight schedule, and hence there is a risk that no time will be reserved to observe the video recording of the encounter of the student with the patient. In fact,

it is known that observation in clinical practice does not regularly take place [17].. This means that the observation and the rating (including the feedback to the student) should be part of the planned daily duties and recognized as an important task. If it’s not possible to observe the students in the clinic in real time, because of busy schedules, the debriefing with the student could also take place during educational hours.

The relatively weak correlation between the ORT and the PERT suggests that clinical reasoning as is performed during an encounter does not necessarily reflect the contents and the quality of the analysis that will be written down afterwards while completing a post encounter form. This problem has been recognized before: judgment of clinical problem-solving skills by observation of medical students poorly correlates with the judgment of their written analysis [18]. Thus, both assessment methods are not exchangeable. It is clear that quite different skills are involved in showing how to perform clinical reasoning during encounter with a patient and how to put the analysis on paper in a medical record afterwards. For clinical teachers this means that both elements of clinical practice need attention in the guidance of medical students, and that different instruments are needed to assess these two. Because of the study setting and for practical reasons, the assessors gave no direct feedback to the students. When used in clinical practice, combined direct observation and assessment of the post-encounter form will provide an excellent opportunity for meaningful feedback on clinical reasoning.

Our study has some limitations.

First, our study was limited to one clinical encounter per student. Extension to more encounters per student is needed to establish how many encounters should be observed for proper judgment of the clinical reasoning abilities of the student practice.

Ratings on the post-encounter form may also have been influenced by the observed performance of the student during the encounter. This bias is hard to avoid, since the assessor needs to know which clinical information was used for the post encounter form that was recorded by the student.

## Conclusions

In conclusion, this pilot study shows that the PERT and ORT are both valid, reliable and feasible instruments for assessment of medical students’ clinical reasoning in clinical practice during history taking. Six to seven raters are needed for a reliable assessment of such a complex task, which is not uncommon for workplace-based assessment tools. Future studies are needed to explore their reliability and validity when used on a larger scale with more encounters per student.

The observation rating tool for clinical reasoning presented in this paper provides clinical teachers with an instrument to assess the quality of the clinical reasoning during a student’s encounter with a patient. In our opinion this instrument fills a niche and is a first step towards building consensus among clinical teachers and towards more objectivity in the assessment of medical students during their practical learning.

## Supplementary information

**Additional file 1.** ORT

**Additional file 2.** PEF

**Additional file 3.** PERT

## Data Availability

The datasets used and/or analyzed during the current study are available from the corresponding author on reasonable request.

## Abbreviations

PEF: Post-encounter form
PERT: Post-encounter rating tool
ORT: Observation rating tool

## References

[1] ten Cate O, Durning SJ. Understanding Clinical Reasoning from Multiple Perspectives: A Conceptual and Theoretical Overview. In: Ten Cate O, Custers E, Durning SJ, editors. Principles and Practice of Case-based Clinical Reasoning Education: A Method for Preclinical Students. Cham: Springer; 2018. p. 35–46.

[2] Govaerts MJ, Van de Wiel MW, Schuwirth LW, Van der Vleuten CP, Muijtjens AM (2013). Workplace-based assessment: raters’ performance theories and constructs. Adv Health Sci Educ Theory Pract.

[3] Schuwirth LW, van der Vleuten CP (2012). Programmatic assessment and Kane’s validity perspective. Med Educ.

[4] Ilgen JS, Humbert AJ, Kuhn G, Hansen ML, Norman GR, Eva KW (2012). Assessing diagnostic reasoning: a consensus statement summarizing theory, practice, and future needs. Acad Emerg Med Off J Soc Acad Emerg Med.

[5] Ende J (1983). Feedback in clinical medical education. JAMA.

[6] Durning SJ, Artino A, Boulet J, La Rochelle J, Van der Vleuten C, Arze B (2012). The feasibility, reliability, and validity of a post-encounter form for evaluating clinical reasoning. Med Teach..

[7] Pelgrim EA, Kramer AW, Mokkink HG, van den Elsen L, Grol RP, van der Vleuten CP (2011). In-training assessment using direct observation of single-patient encounters: a literature review. Adv Health Sci Educ Theory Pract..

[8] Haring CM, Cools BM, van Gurp PJM, van der Meer JWM, Postma CT (2017). Observable phenomena that reveal medical students’ clinical reasoning ability during expert assessment of their history taking: a qualitative study. BMC Med Educ.

[9] Hintze J. Power analysis sample size system (PASS) quick start manual: Kaysville, Utah USA: NCSS; 2011.

[10] Cook DA, Zendejas B, Hamstra SJ, Hatala R, Brydges R (2014). What counts as validity evidence? Examples and prevalence in a systematic review of simulation-based assessment. Adv Health Sci Educ Theory Pract..

[11] Mushquash C, O'Connor BP (2006). SPSS and SAS programs for generalizability theory analyses. Behav Res Methods.

[12] Mortaz Hejri S, Jalili M, Masoomi R, Shirazi M, Nedjat S, Norcini J. The utility of mini-Clinical Evaluation Exercise in undergraduate and postgraduate medical education: A BEME review: BEME Guide No. 59. Med Teach. 2019:1–18. 10.1080/0142159X.2019.1652732 31524016

[13] Margolis MJ, Clauser BE, Cuddy MM, Ciccone A, Mee J, Harik P (2006). Use of the mini-clinical evaluation exercise to rate examinee performance on a multiple-station clinical skills examination: a validity study. Acad Med.

[14] van der Vleuten CP, Schuwirth LW, Scheele F, Driessen EW, Hodges B (2010). The assessment of professional competence: building blocks for theory development. Best Pract Res Clin Obstet Gynaecol.

[15] Norcini J, Anderson MB, Bollela V, Burch V, Costa MJ, Duvivier R (2018). 2018 consensus framework for good assessment. Med Teach.

[16] Cook DA, Dupras DM, Beckman TJ, Thomas KG, Pankratz VS (2009). Effect of rater training on reliability and accuracy of mini-CEX scores: a randomized, controlled trial. J Gen Intern Med.

[17] Pulito AR, Donnelly MB, Plymale M, Mentzer RM (2006). What do faculty observe of medical students' clinical performance?. Teach Learn Med..

[18] Woolliscroft JO, Calhoun JG, Beauchamp C, Wolf FM, Maxim BR (1984). Evaluating the medical history: observation versus write-up review. J Med Educ.

