# Supplementary material for: Validity, reliability and feasibility of a new observation rating tool and a post encounter rating tool for the assessment of clinical reasoning skills of medical students during their internal medicine clerkship: a pilot study
Source: BMC Med Educ. 2020 Jun 19;20:198. doi: 10.1186/s12909-020-02110-8 (PMC7304120; doi:10.1186/s12909-020-02110-8)
Supplement: Supplementary file 1 — Additional file 1. ORT [file 12909_2020_2110_MOESM1_ESM.doc]

# Observation rating tool for clinical reasoning

| **The student:** | **A B C D E** | | | | | **The student:** |
| --- | --- | --- | --- | --- | --- | --- |
| Does not take the lead in the conversation | O | O | O | O | O | Is taking the lead in the conversation |
| does not respond to relevant information | O | O | O | O | O | recognizes and responds to relevant information |
| does not ask the patient for specification of relevant symptoms | O | O | O | O | O | Asks the patient to specify relevant symptoms. |
| Asks questions merely in a pre-learned order | O | O | O | O | O | Asks specific questions pointing to pathophysiologic thinking. |
| skips from one subject to the other | O | O | O | O | O | puts questions in al logical order |
| does not demonstrate diagnostic thinking by making summaries | O | O | O | O | O | demonstrates by making summaries differential diagnostic thinking |
| and patient are talking past each other | O | O | O | O | O | Is checking whether the patient is understood well |
| body language shows signs of differential diagnostic thinking | O | O | O | O | O | body language did not show signs of differential diagnostic thinking |
| did not collect enough data to be able to make a correct differential diagnosis | O | O | O | O | O | collected enough data to be able to make a correct differential diagnosis |
| was not efficient in collecting data | O | O | O | O | O | was efficient in collecting data |
| lets repetition occur in the conversation | O | O | O | O | O | does not let repetition occur in the conversation |

A: Left statement is (almost) entirely applicable

B: Left statement is mainly applicable

C: Both statements are equally applicable

D: Right statement is mainly applicable

E: Right statement is (almost) entirely applicable
