# Supplementary material for: Validity, reliability and feasibility of a new observation rating tool and a post encounter rating tool for the assessment of clinical reasoning skills of medical students during their internal medicine clerkship: a pilot study
Source: BMC Med Educ. 2020 Jun 19;20:198. doi: 10.1186/s12909-020-02110-8 (PMC7304120; doi:10.1186/s12909-020-02110-8)
Supplement: Supplementary file 2 — Additional file 2. PEF [file 12909_2020_2110_MOESM2_ESM.docx]

**Post Encounter Form for clinical reasoning**

**Summary statement.** *Summarize the most important historical features in this patient’s presentation*:

……………………………………………………………………………………………………………………………………………………………

……………………………………………………………………………………………………………………………………………………………

……………………………………………………………………………………………………………………………………………………………

……………………………………………………………………………………………………………………………………………………………

**Problem list.** *Point out the three most important problems. Start with the main problem*

1 **Main problem:** ………………………………………………………………………………….

2 …………………………………………………………………………………………………………..

3 ……………………………………………………………………………………………………………..

**Differential diagnosis.** *list the three most likely diagnoses that would explain the main problem you have listed. Start with the most likely diagnosis.*

1 **Most likely diagnosis**: ………………………………………………………………………………………

2………………………………………………………………………………………………………………………………………….

3………………………………………………………………………………………………………………………………………….

**Supporting data for de most likely diagnosis.** *Provide three key history facts that support the most likely diagnosis*

1……………………………………………………………………………………………………………………………………………………….

2……………………………………………………………………………………………………………………………………………………….

3……………………………………………………………………………………………………………………………………………………….

**Physical examination plan.** *Provide three physical examination items you consider important to perform derived from your differential diagnosis.*

1………………………………………………………………………………………………………………………………………………………

2………………………………………………………………………………………………………………………………………………………

3………………………………………………………………………………………………………………………………………………………
