# Supplementary material for: Validity, reliability and feasibility of a new observation rating tool and a post encounter rating tool for the assessment of clinical reasoning skills of medical students during their internal medicine clerkship: a pilot study
Source: BMC Med Educ. 2020 Jun 19;20:198. doi: 10.1186/s12909-020-02110-8 (PMC7304120; doi:10.1186/s12909-020-02110-8)
Supplement: Supplementary file 3 — Additional file 3. PERT [file 12909_2020_2110_MOESM3_ESM.docx]

# Post encounter rating tool for clinical reasoning

|  | **A B C D E** | | | | |
| --- | --- | --- | --- | --- | --- |
| **Summary statement** | O | O | O | O | O |
| **Problemlist** | O | O | O | O | O |
| **Differential diagnosis** | O | O | O | O | O |
| **most likely diagnosis with supporting data** | O | O | O | O | O |
| **physical examination plan** | O | O | O | O | O |

A=poor

B=moderate

C=adequate

B=good

C=outstanding
